# Identification of the Gene Responsible for Lignin-Derived Low-Molecular-Weight Compound Catabolism in *Pseudomonas* sp. Strain LLC-1

**DOI:** 10.3390/genes11121416

**Published:** 2020-11-27

**Authors:** Jun Hirose, Ryusei Tsukimata, Munetoshi Miyatake, Haruhiko Yokoi

**Affiliations:** Faculty of Engineering, University of Miyazaki, Miyazaki 889-2192, Japan; tsuky.0111@docomo.ne.jp (R.T.); t0g205u@cc.miyazaki-u.ac.jp (M.M.); yokoi@cc.miyazaki-u.ac.jp (H.Y.)

**Keywords:** benzoylformic acid, *bzf* gene, lignin, mandelic acid, *Pseudomonas*, vanillin, vanillin dehydrogenase

## Abstract

*Pseudomonas* sp. strain LLC-1 (NBRC 111237) is capable of degrading lignin-derived low-molecular-weight compounds (LLCs). The genes responsible for the catabolism of LLCs were characterized in this study using whole-genome sequencing. Despite the close phylogenetic relationship with *Pseudomonas putida*, strain LLC-1 lacked the genes usually found in the *P. putida* genome, which included *fer,* encoding an enzyme for ferulic acid catabolism, and *vdh* encoding an NAD^+^-dependent aldehyde dehydrogenase specific for its catabolic intermediate, vanillin. Cloning and expression of the 8.5 kb locus adjacent to the *van* operon involved in vanillic acid catabolism revealed the *bzf* gene cluster, which is involved in benzoylformic acid catabolism. One of the structural genes identified, *bzfC*, expresses the enzyme (BzfC) having the ability to transform vanillin and syringaldehyde to corresponding acids, indicating that BzfC is a multifunctional enzyme that initiates oxidization of LLCs in strain LLC-1. Benzoylformic acid is a catabolic intermediate of (*R,S*)-mandelic acid in *P. putida*. Strain LLC-1 did not possess the genes for mandelic acid racemization and oxidation, suggesting that the function of benzoylformic acid catabolic enzymes is different from that in *P. putida*. Genome-wide characterization identified the *bzf* gene responsible for benzoylformate and vanillin catabolism in strain LLC-1, exhibiting a unique mode of dissimilation for biomass-derived aromatic compounds by this strain.

## 1. Introduction

Microorganisms capable of catabolizing aromatic compounds play an important role in degrading aromatic biomass compounds in the environment, major components of which are lignin-derived low-molecular-weight compounds (LLCs) produced by lignin depolymerization. They are composed of aldehydes, ketones, and organic acids. Aromatic LLCs are typically catabolized through the upper and lower ring fission pathways, where the aromatic rings are cleaved and subsequent metabolites enter the central carbon metabolism [1].

Many genes coding for the LLC metabolic pathways have been isolated and characterized. The upper and lower ring fission pathways are encoded by different loci and are controlled by independent transcriptional regulators [2,3]. The genes coding for lower pathways, such as potocatechate, benzoate, and gentisate, are ubiquitous and well conserved in many aerobic bacteria [2]. Recent advances in DNA sequencing technology have enabled the identification of additional genes involved in LLC catabolism; however, most of these remain to be studied in terms of their function and regulation.

Vanillin is one of the major components of LLCs. Both bio-production [4,5] and bio-degradation [6] of vanillin are of interest. Vanillin itself is a flavor that is used in the food industry. Many attempts have been made to produce dicarboxylic bio-plastic monomer compounds from LLC using engineered bacteria [7,8,9]. Several pathways involving bacterial catabolism of vanillin have been reported. Vanillin is a catabolic intermediate of eugenol [10,11], isoeugenol [12], or ferulic acid [13,14] in *Pseudomonas* and other bacteria. They are transformed into vanillin via ligation by coenzyme A and subsequent cleavage of the propanoic side chain. The vanillin catabolic pathway is initiated by oxidation to vanillic acid and undergoes monooxygenation to form protocatechuate. This is followed by dioxygenation to form ring-cleavage intermediate compounds that enter the tricarboxylic acid cycle via the *β*-ketoadipate pathway [2].

We identified a gram-negative aerobic bacterial isolate, *Pseudomonas* sp. strain LLC-1 (here referred to as strain LLC-1) using enrichment culture in a medium containing LLCs and inorganic salts [15]. This bacterial strain can utilize vanillin and vanillic acid as sole carbon sources, and co-metabolizes syringaldehyde, *o*-vanillin, and isovanillin. Here, we present the identification and characterization of a gene responsible for the catabolic pathway of LLCs using whole-genome sequencing.

## 2. Materials and Methods

### 2.1. Bacterial Strains, Plasmid, and Cultivation

The bacterial strains used in this study are presented in Table 1. *Pseudomonas* sp. strain LLC-1 (NBRC 111237) was isolated from soil in Miyazaki, Japan [15]. *P. putida* NBRC 14164 was obtained from the Biological Resource Center of the National Institute of Technology and Evaluation (NBRC), Tokyo, Japan. For the growth of *Pseudomonas* strains, a basal salts medium (BSM) containing (in grams per liter) K_2_HPO_4_, 4.3; KH_2_PO_4_, 3.4; (NH_4_)_2_SO_4_, 2.0; MgCl_2_·6H_2_O, 0.34; MnCl_2_·4H_2_O, 0.001; FeSO_4_·7H_2_O, 0.0006; CaCl_2_·2H_2_O, 0.026; and Na_2_MoO_4_·2H_2_O, 0.002 (pH 7.0) was used. BSM was supplemented with 0.1% (*w*/*v*) of various aromatic compounds. The bacterial strains were grown with shaking (120 rpm) at 30 °C and the growth was monitored by optical density measurements at 600 nm. For DNA isolation or freezing stock preparation, Luria–Bertani (LB) medium (Bacto Trypton, 10 g; yeast extract, 5 g; and NaCl, 10 g per liter, pH 7.0) was used. The *Escherichia coli* cells were grown with shaking (160 rpm) at 37 °C in Terrific broth (TB) medium (1.2% Bacto Tryptone, 2.4% yeast extract, 0.5% glycerol, 0.17 M KH_2_PO_4_-0.72 M K_2_HPO_4_ 10%(*v*/*v*)) supplemented with ampicillin sodium (100 μg/mL). Isopropyl-β-D-thiogalactpyranoside (IPTG) was added to a final concentration of 0.2 mM to induce gene expression under the control of the *lac* promoter in the plasmid vector.

### 2.2. Genome Sequencing and Computational Analysis

The whole genome sequence of strain LLC-1 was determined as previously reported [16]. The reads obtained by the two systems were assembled using Newbler v.2.8 (Roche, Basel, Switzerland). The genome sequences were annotated using the NCBI Prokaryotic Genome Annotation Pipeline (PGAP) [17] and Rapid Annotations using Subsystems Technology (RAST) server v.2.0 [18]. Identification of coding genes was performed using BLAST and BLASTX searches [19]. Sequence comparisons were performed using EasyFig v.2.1 [20], and a map was generated using drawGeneArrows3. The 16S rRNA gene sequences were aligned computationally using the ClustalW algorithm, as described. Phylogenetic trees were generated using the neighbor-joining method with the MEGA X program [21]. The trees were evaluated through bootstrap resampling (500 replicates).

### 2.3. Cloning and Expression of bzf Gene Cluster

DNA isolation, Southern blot analysis, PCR, DNA sequencing, and other DNA manipulations were performed according to standard procedures as described [23]. A sequence encoding the *bzfC* gene (1.58 kb) was amplified by PCR using primers VSDH-6 (5′-GGCCATTGCCCTTATCCAGGTG-3′) and VSDH-7 (5′-GTGGTCGAAAGGCTGCATGAATG-3′), and inserted into pMD20, using the Mighty TA-cloning Kit (Takara Bio Inc., Kusatsu, Japan), yielding the plasmid pMDBzfC. A 1.58 kb *Xba*I-*Bam*HI fragment was excised from pMDBzfC and inserted into pBluescriptKS+ using the Ligation-Convenience Kit (Nippon Gene, Tokyo, Japan), yielding the plasmid pKSBzfC. An 8.5 kb fragment was amplified from LLC-1 genomic DNA using the primers VSDH-13 (5′-GGTATCGATAAGCTTGATCCAGTCATGAGTGGTTTTCCAGG-3′) and VSDH-14 (5′-GGGCTGCAGGAATTCGATCTGTTGGCTTGTTCGGGCAATAC-3′) and PrimeSTAR GXL DNA Polymerase (Takara Bio Inc.). The PCR fragment was treated with Cloning Enhancer and inserted into pBluescriptKS+, digested with *Eco*RV, using an In-Fusion^®^ HD Cloning Kit (Clontech Laboratories, Mountain View, CA, USA) to yield the plasmid pKSBzf1. The consistency of the sequence of each plasmid with the genomic sequence was checked by DNA sequencing. All plasmids were transformed into *E. coli* JM109 cells.

### 2.4. Metabolism of LLCs by Bacterial Strains

*E. coli* cells grown in TB were harvested by centrifugation and suspended to a turbidity of 2.0 at 600 nm in 50 mM phosphate buffer (pH 7.5) containing 20 mM glucose. Cell suspensions (20 mL) in 200 mL flasks were incubated at 30 °C on a rotary shaker (120 rpm) in the presence of substrates (1 mM). After 12 h incubation, the pH of the sample was adjusted to 2.0–3.0 with 1 M HCl and then extracted with 1.5-fold volumes of ethyl acetate. The organic extracts were dried over anhydrous sodium sulfate and concentrated under vacuum at 37 °C. The residual extracts were dried with anhydrous sodium sulfate and concentrated using a rotary evaporator. The samples were dissolved in methanol and subsequently subjected to gas chromatography-mass spectrometry (GC-MS) analysis.

### 2.5. Analytical Procedures

GC-MS analysis was performed on a Shimadzu model QP-5050A (Shimadzu Co., Kyoto, Japan) platform equipped with a Phenomenex ZB-5 capillary column (inside diameter: 0.25 mm; film thickness: 0.25 mm; length: 30 m) (Phenomenex, Torrance, CA, USA). The column temperature was programmed at 80 °C for 2 min, from 80 to 200 °C at 15.0 °C/min, from 200 to 240 °C at 10.0 °C/min. For trimethylsilyl (TMS) derivatization, samples were dried under vacuum and treated with 30 μL of *N*-Methyl-*N*-trimethylsilyltrifluoroacetamide (MSTFA) and 7.5 μL of pyridine at room temperature for 30 min. Oxygen consumption during the metabolism of aromatic compounds by bacteria was measured as described previously [15], and dissolved dioxygen was monitored using a Clark-type polarographic O_2_ electrode (Yellow Springs Instruments Model 5300A, Yellow Springs, OH, USA). To achieve this, 3 mL of cell suspension in a chamber was incubated at 30 °C on a magnetic stirrer in the presence of the substrate.

## 3. Results

### 3.1. Phylogenetic Analysis of Strain LLC-1

**Figure 1:**
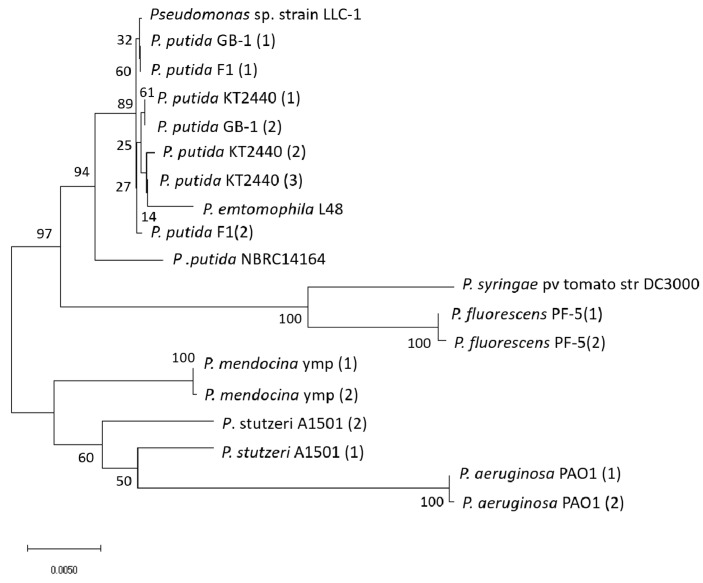
16S rRNA phylogenetic tree of *Pseudomonas* strains (GenBank/ENA/DDBJ Accession Number); *Pseudomonas* sp. strain LLC-1 (NZ_LUVY00000000), *P. putida* KT2440 (AE015451), *P. putida* F1 (CP000712), *P. putida* GB-1 (CP000926), *P. putida* NBRC14164 (AP013070), *P. aeruginosa* PAO1(NC_002516), *P. mendocina* ymp (CP000680), *P. fluorescens* Pf-5 (CP000076), *P. stutzeri* A1501 (NC_009434), *P. syringae* pv tomato str DC3000 (AE016853) and *P. emtomophila* L48 (CT573326). The numbers in parentheses indicate variants found in one strain. Multiple sequence alignment outputs of the 16S rRNA sequence (1528 bp) were used to generate neighbor-joining phylogenetic trees using MEGA X [21]. The bar indicates expected nucleotide substitutions per site. Numbers indicate the percentage occurrence of the branch in the bootstrapped trees of 500 replicates.

A phylogenetic tree of strain LLC-1 and various *Pseudomonas* species is shown in Figure 1. The 16S rRNA of strain LLC-1 was almost identical to that of *P. putida* GB-1 and *P. putida* F1 (99.9%), with one or two base substitutions, compared with that of *P. putida* GB-1 or *P. putida* F1. From the topology of the phylogenetic tree, strain LLC-1 clustered clearly with the *P. putida* species (Figure 1), with the exception of *P. putida* NBRC14164, which is distant from other *P. putida* strains [22]. Comparison of the genome sequences available in the RAST data sets revealed that *P. putida* GB-1 is the closest neighbor of strain LLC-1, followed by *P. putida* F1 [16]. These results are consistent with the 16S rRNA phylogenetic analysis. The 16S rRNA of other species exhibited < 99.4% similarity to that of strain LLC-1, showing a distant relationship with strain LLC-1.

### 3.2. Detection of Catabolic Genes for Aromatic Compounds in Strain LLC-1

Catabolic genes for various aromatic acids were identified from the draft genome sequence of strain LLC-1: anthranilate catabolic *atn* gene, benzoate catabolic *ben* gene, catechol catabolic *cat* gene, 4-hydroxybenzoic acid catabolic *pob* gene, 4-hydroxyphenylacetic acid catabolic, *hpa* gene, homogentisate catabolic *hmg* gene, phenylacetate catabolic *paa* gene, protocatechuate catabolic *pca* gene, and vanillate catabolic *van* genes were found. Aromatic compound catabolic genes found in the genome sequence of strain LLC-1 are shown in Table S1. Protocatechuate, homogentisate, and benzoate are known as key intermediates of funneling pathways for aromatic ring degradation. The *van* gene encodes a two-component vanillate-*O*-demethylase (VanAB), and a regulatory component involved in oxidative demethylation of vanillic acid. The *pca* gene encodes *β*-ketoadipate pathway enzymes following ring-cleavage reactions, which subsequently enter the tricarboxylic acid cycle.

**Figure 2:**
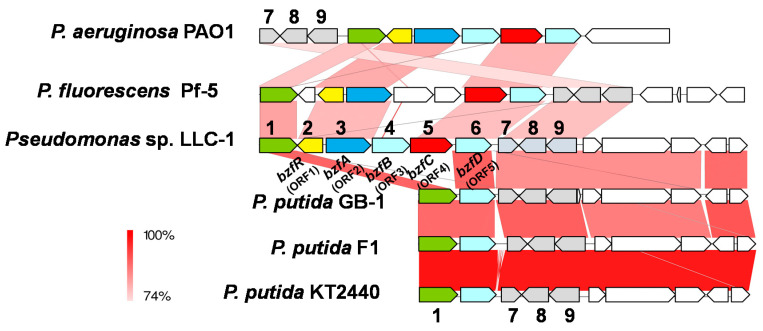
Physical map of benzoylformate catabolic *bzf* gene cluster and vanillate cetabolic *van* operon of *Pseudomoas* sp. strain LLC-1 and other pseudomonas strains. 1, *vanK*; 2, *bzfR* (ORF1); 3, *bzfA* (ORF2); 4, *bzfB* (ORF3); 5, *bzfC* (ORF4); 6, *bzfD* (ORF5); 7, *vanR*; 8, *vanA*; 9, *vanB*;. ORFs with conserved sequence (>70% identity with those of LLC-1 within *bzf* gene cluster or *van* operon) are filled with the same color. The shading in pink to red shows the identity (74–100%) of the gene clusters as indicated on the left of the figure.

Despite the close phylogenetic relationship to *P. putida*, strain LLC-1 lacked certain genes usually found in the *P. putida* genome; strain LLC-1 does not possess the *fer* gene, which encodes the ferulic acid metabolic pathway widely distributed in the genome of *P. putida* [14], and the *vdh* gene encoding an NAD^+^-dependent aldehyde dehydrogenase that specifically oxidizes its catabolic intermediate, vanillin. In addition, strain LLC-1 was deficient in genes encoding the isoeugenol (*iem*) catabolic pathways detected in the genome of a minor group of *P. putida* [12]. We attempted to search the draft genome sequence of the LLC-1 strain for a gene encoding vanillin dehydrogenase, which converts vanillin to vanillic acid. Annotation by RAST detected 47 open reading frames (ORFs) identified as aldehyde dehydrogenase. Two of these were benzaldehyde dehydrogenases. A BLASTX search was performed on one of the open reading frames present in the vicinity of the *van* operon encoding vanillate demethylase. The ORF showed 65% identity with the *vdh* gene encoding vanillin dehydrogenase of *P. putida* strain IE27 [12], which is a component of the isoeugenol catabolic gene cluster. The ORF identified as benzaldehyde dehydrogenase (ORF4) was adjacent to the LysR family of transcriptional regulators (ORF1), benzoylformate decarboxylase (ORF2), putative major facilitator superfamily (MFS) transporters that are related to benzoate transporter BenK of *Acinetobacter* sp. [24] (ORF3), and outer membrane OprD family porin protein (ORF5), which is related to the putative phenylacetic acid uptake porin PhaK of *P. putida* [25] (Figure 2). A BLASTX search showed that ORF2 exhibited 84% and 63% identity at the amino acid level to thiamine diphosphate-dependent benzoylformate decarboxylase from *P. fluorescens* (PfBFDC) [26] and *P. putida* (MdlC) [27], respectively. MdlC is known to be involved in mandelic acid metabolism in mandelic acid-degrading *P. putida.* From these results, it was presumed that this gene cluster is involved in the transport and catabolism of benzoylformic acid. The gene clusters encoded by these five ORFs were named *bzfR, bzfA, bzfB, bzfC,* and *bzfD,* as shown in Figure 2.

Genes closely related to the *bzf* gene cluster in *P. fluorescens* Pf-5 [26] and *P. aeruginosa* [28] have been reported, including benzoylformate decarboxylase, MFS transporter, aldehyde dehydrogenase, and porin protein. The gene organization of the regions corresponding to *bzfRABCD* of strain LLC-1 is shown in Figure 2. The five components of the *bzf* gene cluster showed 78–81% identity with those of *P. fluorescens* Pf-5, and 73–80% identity with those of *P. aeruginosa* PAO1, respectively, with the exception of *bzfB* whose homolog was not found for Pf-5. From the genome sequences of *P. putida* F1, *P. putida* GB-1, and *P. putida* KT2440, a gene cluster showing identity with the *bzf* gene cluster was not detected (Figure 2).

### 3.3. Biotransformation of LLCs by E.coli Cells Expressing bzf Gene Cluster

**Figure 3:**
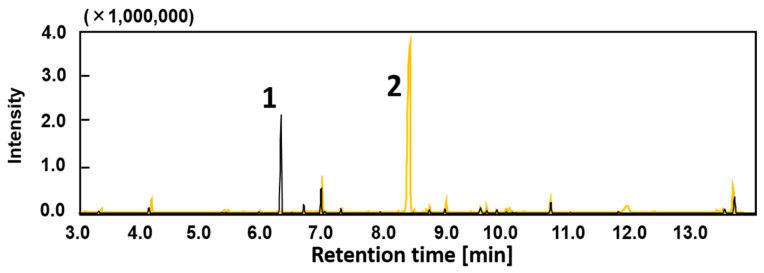
Conversion of benzoylformate by *E. coli* cells carrying pKSBzf1 (black line) or *E. coli* cells carrying pBluescriptKS+ (yellow line). Peak 1, benzoic acid; 2, benzoylformic acid. The products were derivatized with MSTFA and identified comparing the retention time and mass spectra of authentic trimethylsilylated standards. *m*/*z* values of each peak are listed in Supplemental Table S2.

We cloned the *bzfABCD* gene cluster (8.5 kb locus adjacent to the *van* operon) and expressed it in *E. coli* to investigate the function of the *bzf* gene products. The results of GC-MS analysis of the reaction products after the degradation of various aromatic carboxylic acids by resting cells of recombinant *E. coli* JM109 (pKSBzf1) expressing the *bzfABCD* gene are shown in Figure 3. When benzoylformic acid was used as a substrate, in the vector control obtained by incubating *E. coli* carrying pBluescript KS+, the degradation of benzoylformic acid did not occur. In contrast, *E. coli* cells carrying pKSBzf1 completely converted benzoylformic to benzoic acid. The *bzf* gene cluster was suggested to be involved in benzoylformic acid catabolism. Although BzfA is presumed to decarboxylate vanillic acid to guaiacol, the reaction products vanillic acid and the by-product vanillyl alcohol were detected, whereas guaiacol was not detected when *E. coli* cells carrying pKSbzf1 were incubated with vanillin. Therefore, decarboxylation by BzfA is likely to be benzoylformatic acid-specific.

**Figure 4:**
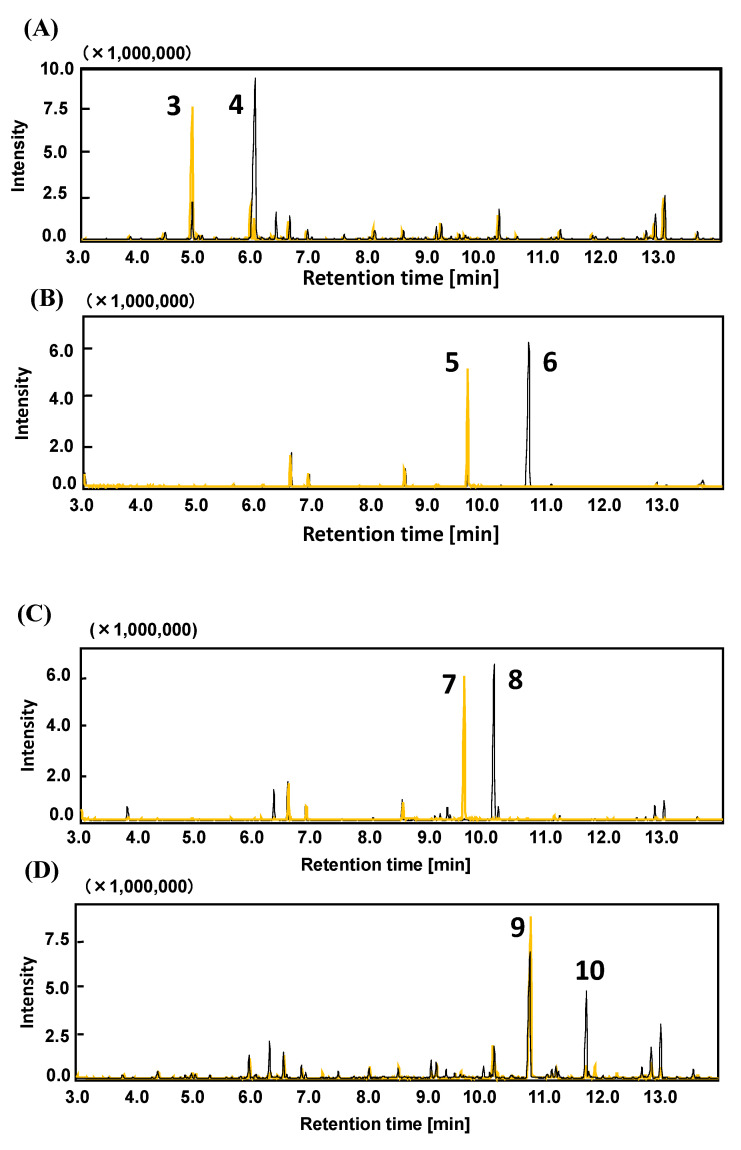
Conversion of bezylaldehyde (**A**), vanillin (**B**), isovanillin (**C**), and syringaldehyde (**D**) by *E. coli* cells carrying pKSBzfC (black line) or *E. coli* cells carrying pBluescriptKS+ (yellow line). Peak 3, benzyl alcohol; 4, benzoic acid; 5, vanillyl alcohol; 6, vanillic acid; 7, isovanillyl alcohol, 8; isovanillic acid; 9, syringic alcohol; 10, syringic acid. The products were derivatized with MSTFA and identified by comparing the retention time and mass spectra of authentic standards. *m*/*z* values of each peak are listed in Supplemental Table S2.

*BzfC*, a structural gene of the *bzf* gene cluster, has been identified as an NAD^+^-dependent dehydrogenase. It is proposed to be involved in the oxidation of benzaldehyde produced by the decarboxylation of benzoylformic acid. Strain LLC-1 lacked the ferulic acid catabolic *fer* gene, and the *vdh* gene encoding an NAD^+^-dependent aldehyde dehydrogenase specific for vanillin. It was confirmed that *bzfC* shows 65% identity with the *vdh* gene, encoding the vanillin dehydrogenase component of the isoeugenol catabolic gene cluster in *P. putida* IE27, as described above. Therefore, we assumed that *bzfC* is involved in the oxidization of both benzaldehyde and vanillin. We investigated the transformation of benzaldehyde, vanillin, isovanillin, and syringaldehyde in *E. coli* cells expressing the *bzfC* gene. In the vector control, *E. coli* carrying pBluescript KS+ reduced all aldehydes investigated to the corresponding alcohol completely (Figure 4), a reaction assumed to be catalyzed by benzaldehyde reductase from *E. coli* host cells [29]. After the conversion of benzylaldehyde and vanillin for 12 h, in reaction mixtures containing recombinant *E. coli* carrying plasmid pKSBzfC, benzoic acid, and vanillic acid instead of benzyl alcohol and vanillyl alcohol were detected (Figure 4A,B). The conversion of isovanillin by *bzfC*-expressing *E. coli* resulted in the formation of the corresponding carboxylic acids, whereas part of syringaldehyde was transformed into syringic acid (Figure 4C,D). From these results, it was confirmed that oxidization by aldehyde dehydrogenase encoded by *bzfC* (*BzfC*) is not limited to benzaldehyde produced by benzoylformic acid decarboxylization, and is expanded to vanillin, isovanillin, and syringaldehyde.

### 3.4. Induction of Benzoylformic Acid Metabolism

**Figure 5:**
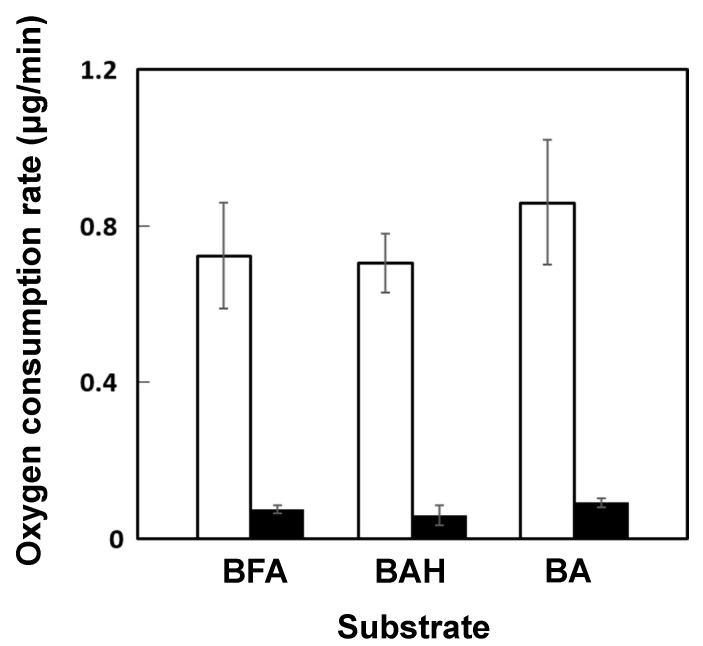
Oxygen consumption by strain LLC-1 grown with benzoylformic acid (white box) or pyruvic acid (black box). The cell suspensions (at OD_600_ of 2.0) were incubated with 150 μM benzoylformic acid (BFA), benzaldehyde (BAH), or benzoic acid (BA). Dissolved dioxygen in a chamber was monitored as described in Materials and Methods. Experiments were performed in triplicates, and standard deviations are displayed as error bars.

The metabolic ability of resting cells of strain LLC-1 grown in a medium containing benzoylformic acid or pyruvic acid as the sole carbon source was compared by measuring oxygen consumption rates (Figure 5). Strain LLC-1 grown on benzoylformic acid as the sole carbon source showed a higher oxygen uptake rate in samples incubated with benzoylformate, benzaldehyde, and benzoic acid, compared to cells grown on pyruvate as a carbon source. This result indicated that strain LLC-1 can metabolize benzoylformic acid and its catabolic intermediate benzaldehyde and benzoic acid more efficiently under induction by benzoylformic acid.

### 3.5. Growth on Mandelic Acid

**Figure 6:**
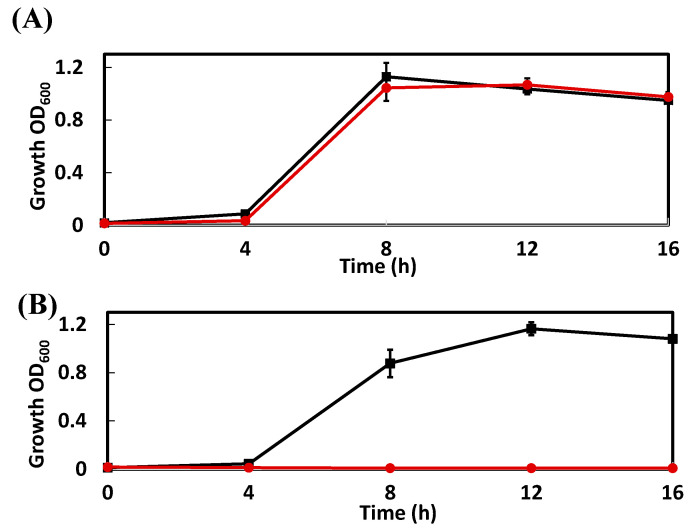
Growth of strain LLC-1 and *P. putida* NBRC 14164 in culture medium containing benzoylformic acid (**A**) and (*R.S*)-mandelic acid (**B**). The circles (red) represent the growth profile of strain LLC-1 and squares (black) represent the growth profile of *P. putida* NBRC 14164. Experiments were performed in triplicates, and standard deviations are displayed as error bars.

Benzoylformic acid, a substrate for benzoylformate decarboxylase encoded by *bzfA*, is known to be a catabolic intermediate in the mandelate pathway encoded by the *mdl* gene of *P. putida* [27]. The *bzfA* genes showed 63% identity at the amino acid level with the *mdlC* genes, but the homologous gene for *mdlA* encoding mandelate racemase and the *mdlB* gene encoding (*S*)-mandelate dehydrogenase were not present in the genome of strain LLC-1. The growth of strain LLC-1 and the mandelic acid-degrading bacterial strain, *P. putida* NBRC 14164, in a medium containing (*R,S*)-mandelic acid or benzoylformic acid as the sole carbon source were examined (Figure 6). Strain LLC-1 and *P. putida* NBRC 14164 were able to grow on benzoylformic acid as a sole carbon source, but strain LLC-1 was not able to grow on mandelic acid. From these results, it was concluded that strain LLC-1 does not produce mandelate racemase and mandelate dehydrogenase, which transforms mandelate to benzoylformic acid.

## 4. Discussion

In this study, the draft genome sequence of strain LLC-1 was used to characterize the *bzf* gene cluster involved in the catabolism of LLCs. The sequence of the 16S rRNA gene indicated that strain LLC-1 is phylogenetically close to *P. putida* (Figure 1), but the overall genomic structure exhibited variation between strain LLC-1 and *P. putida.* LLC-1 does not possess the *fer* gene, involved in the ferulic acid metabolic pathway widely distributed in the genome of *P. putida* [11], and the *vdh* genes encoding a dehydrogenase that specifically oxidizes its catabolic intermediate, vanillin. In addition, strain LLC was deficient in genes encoding the isoeugenol (*iem*) catabolic pathways detected in the genome of a sub-group of *P. putida* [12], as well as the *mdl* gene encoding the mandelic acid degradation pathway [22,27]. On the other hand, strain LLC-1 carried the *bzf* gene cluster encoding benzoylformic acid catabolic enzymes and transporters that do not exist in the *P. putida* genome.

**Figure 7:**
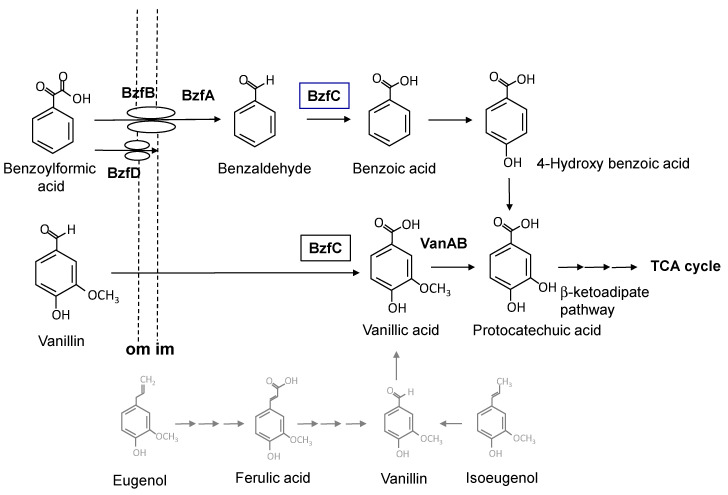
Putative metabolic pathways of benzoylformic acid and vanillin in strain LLC-1. Enzymes: BzfA, benzoylformate carboxylase; BzfC, benzaldehyde dehydrogenase/vanillin dehydrogenase; VanAB, vanillin-*O*-demethylase. Transporters: BzfB, BenK-like MFS transporter; BzfD, PhaK-like outer membrane porin. Structures: om, outer membrane; im, inner membrane. BzfC which convert both benzaldehyde and vanillin was highlighted within boxes. Catabolic pathways for eugenol, ferulic acid and isoeugenol of *P. putida* which are described in the ‘Introduction’ section, are represented in gray.

In this study, the *bzf* gene cluster of strain LLC-1 was shown to be involved in the conversion of benzoylformic acid to benzoic acid (Figure 7). Benzoic acid can be degraded to tricarboxylic acid cycle intermediates via the *β*-ketoadipate pathway encoded by the *pca g*ene in the genome of strain LLC-1 (Table S1) [30]. The first ORF at the end of the *bzf* gene cluster was identified as a transcriptional regulator (*bzfR*). Benzoylformic acid-metabolizing enzymes were induced by benzoylformic acid in strain LLC-1 (Figure 5), and *bzfR* is a putative transcriptional regulator of benzoylformic acid catabolic enzymes. We previously reported that vanillin-catabolizing enzymes are not induced by vanillin in strain LLC-1 [15]. This may be due to poor interaction between the putative transcriptional regulator BzfR and vanillin or its metabolite, vanillic acid. This transcriptional regulator is likely to be benzoylformic acid-specific. The second ORF (*bzfA*) in the *bzf* gene cluster showed 84% or 63% identity at the amino acid level with thiamine diphosphate-dependent benzoyldormate decarboxylase from *P. putida* (MdlC) or *P. fluorescence* (PfBFDC), respectively [26,27]. Three-dimensional structures of both MdlC (PDB entry code: 1BFD) [31] and PfBFDC (6QSI) [26] are available. They are enzymes whose reaction mechanisms have been precisely investigated based on their structure [32,33]. The results of the biotransformation study (Figure 3) and comparison of sequences with these known benzoylformate decarboxylases (Figure 2) supported the hypothesis that BzfA is an enzyme that decarboxylates benzoylformic acid. The fourth ORF (*bzfC*) located between *bzfB* and *bzfD* showed 65% identity at the amino acid level with the *mdlD* gene encoding benzaldehyde dehydrogenase of *P. putida* [27]. It is obvious that *bzfA* and *bzfC* are homologous to *mdlA* and *mdlC*, respectively. However, the overall structures of the *bzf* and *mdl* gene clusters are completely different. *MdlA* and *mdlC* are encoded in the opposite direction to each other and are located on a clearly different transcription unit. It is presumed that the *mdl* gene cluster underwent reorganization during evolution. Transporter membrane proteins are usually required for the uptake of aromatic carboxylic acids into cells through the cell membrane [34]. The BenK-like MFS transporter encoded by the third ORF, *bzfB*, and the PhaK-like outer membrane OprD family porin protein encoded by the fifth ORF, *bzfD*, are putative transporters specific for benzoylformic acid (Figure 7). They may transport benzoylformic acid in strain LLC-1, as well as in recombinant *E. coli* expressing the *bzf* gene cluster. On the other hand, vanillin is proposed to be incorporated via simple diffusion in strain LLC-1 and recombinant *E. coli* cells due to its hydrophobic nature [35].

Benzoylformic acid is known to be an intermediate of the mandelic acid catabolic pathway of *P. putuda*. Benzoylformic acid is produced through the isomerization and oxidation of mandelic acid and then undergoes decarboxylation and oxidization to form benzoic acid. Strain LLC-1 did not utilize (*R,S*)-mandelates as a carbon source (Figure 6). In addition, ORFs coding for enzymes involved in the metabolism of mandelic acid was not found. Therefore, the role of benzoylformic acid catabolic enzymes of strain LLC-1 is different from that of *P. putida*, where strain LLC-1 assimilates benzoylformic acid, and not as a metabolic intermediate of other carbon sources. Wei et al. recently studied the role of the benzoylformate decarboxylase of *P. fluorescens* Pf-5 in the degradation of lignin depolymerization fragments. It was confirmed that benzoylformate decarboxylase does not act on guaiacil-glycerol derivatives, an allyl C3 lignin-degrading fragment, and specifically decarboxylates benzoylformic acid or 4-hydroxybenzoylformic acid, which represent aryl C2 substrates [26]. It was proposed that 4-hydroxybenzoylformic acid is formed during the degradation of lignin via oxidation of a keto-aldehyde precursor. Our results provided insight into the role of the benzoylformate pathway encoded by the *bzf* gene cluster of strain LLC-1 in lignin degradation via the formation of 4-hydroxybenzoylformic acid.

The result that *E. coli* expressing the *bzfC* gene oxidized vanillin and syringaldehyde to the corresponding acids indicated that BzfC is a multifunctional enzyme that initiates oxidization of LLCs in strain LLC-1. The substrates of BzfC were not limited to benzaldehyde, which is a catabolic intermediate of benzoylformic acid. This result was consistent with our previous study in which the same substrates were transformed into their corresponding aromatic acids as dead-end products by strain LLC-1 [15]. Alternative use of BfzC in benzoylformate and vanillin catabolism may be due to adaptation to environmental niches. There are two other reasons to explain the role of BzfC in vanillin catabolism. First, the amino acid sequence encoded by the *bzfC* gene showed relatively high identity (65%) with that of vanillin dehydrogenase (Vdh) of *P. putida* IE27 [12]. Second, the *bzf* gene cluster is flanked by the *van* operon, which encodes a downstream vanillate-metabolizing enzyme (*vanAB*) and its transcriptional regulator (*vanR*). Cross-regulation between *bzf* and *van* genes may be feasible when the two genes are located at short distances from each other [36,37].

There are examples of studies that attempted to produce vanillin from ferulic acid by constructing a vanillin dehydrogenase-deficient microbial strain [13,14]. Construction of mutant strains deficient in *bzfB, bzfC,* or *bzfD* will be useful to determine the role of *bzf* in vanillin catabolism and benzoylformic acid transportation.

## 5. Conclusions

In this study, we revealed that BzfC has a wide substrate range and is an important enzyme that initiates the oxidation of aromatic biomass (LLC) in strain LLC-1. Genome-wide characterization demonstrated that the strain LLC-1 catabolized benzoylformic acid and vanillin via a metabolic pathway that is independent of ferulic acid, eugenol, isoeugenol, or mandelic acid catabolic pathways, exhibiting a unique mode of dissimilation for the metabolic pathways related to biomass-derived aromatic compounds.

## Figures and Tables

**Table 1:** Bacterial Strains and Plasmids Used in this Study.

Strain or Plasmid	Relevant Characteristic(s)	Source or Reference
Bacterial strains *Pseudomonas* sp. strain LLC-1 (NBRC 111237)	VNL^+^ MDL^−^	[15,16]
*P. putida* NBRC 14164	MDL^+^	[22]
*E. coli* JM109	*recAl endAl gyrA96 thi hsdRl7 supE44 relA1Δ(lac proAB)* [*F’proAB laclq DM15 traD36*]	Laboratory stock
Plasmid		
pBluescript KS+	*lacZ* Ap^r^	Agilent Technologies/Stratagene
pMD20	*lacZ* Ap^r^	Takara Bio
pMDBzfC	pMD20-*bzfC*	This study
pKSBzfC	pBluescript KS+*-bzfC*	This study
pKSBzf1	pBluescript KS+*-bzfABCD*	This study

VNL, vanillin utilization; MDL, mandelic acid utilization; Ap^r^, ampicillin resistance.

## Supplementary Materials

The following are available online at https://www.mdpi.com/2073-4425/11/12/1416/s1, Table S1: Catabolic genes of aromatic compounds found in the draft genome of strain LLC-1. Table S2: List of data from GC/MS analysis.
