# Supplementary material for: Identification of the Gene Responsible for Lignin-Derived Low-Molecular-Weight Compound Catabolism in Pseudomonas sp. Strain LLC-1
Source: Genes (Basel). 2020 Nov 27;11(12):1416. doi: 10.3390/genes11121416 (PMC7760541; doi:10.3390/genes11121416)
Supplement: Supplementary file 1 [file genes-11-01416-s001.pdf]

Table S1 Catabolic genes of aromatic compounds found in the draft genome of strain LLC-1.

ORF [a]	Gene	Length (bp) [b]	Function [c]	Affiliated catabolic pathway [d]
A0O30_04025	*hmgR*	783	Transcriptional regulator, IclR family	PHE, TYR
A0O30_04030	*hmgA*	1302	Homogentisate 1,2-dioxygenase (EC 1.13.11.5)	PHE, TYR
A0O30_04035	*hmgB*	1293	Fumarylacetoacetase (EC 3.7.1.2)	PHE, TYR
A0O30_04040	*hmgC*	633	Maleylacetoacetate isomerase (EC 5.2.1.2)	PHE, TYR
A0O30_04880	*PcaR*	876	*Pca* regulon regulatory protein PcaR	4HBA,VA
A0O30_04885	*pcaK*	1347	4-Hydroxybenzoate transporter	4HBA,VA
A0O30_04890	*pcaF*	1203	β-ketoadipyl CoA thiolase (EC 2.3.1.-)	4HBA,VA
A0O30_04895	*pcaT*	1290	Dicarboxylic acid transporter PcaT	4HBA,VA
A0O30_04900	*pcaB*	1353	3-Carboxy-cis,cis-muconate cycloisomerase (EC 5.5.1.2)	4HBA,VA
A0O30_04905	*pcaD*	792	β-ketoadipate enol-lactone hydrolase (EC 3.1.1.24)	4HBA,VA
A0O30_04910	*pcaC*	393	4-Carboxymuconolactone decarboxylase (EC 4.1.1.44)	4HBA,VA
A0O30_11560	*pcaJ*	642	3-Oxoadipate CoA-transferase subunit B (EC 2.8.3.6)	4HBA,VA
A0O30_11565	*pcaI*	696	3-Oxoadipate CoA-transferase subunit A (EC 2.8.3.6)	4HBA,VA
A0O30_12090	*hpaR1*	591	Transcriptional regulator, TetR family	4HPA
A0O30_12095	*hpaB*	1170	*p*-Hydroxyphenylacetate hydroxylase C2:oxygenase component	4HPA
A0O30_12100	*hpaC*	930	*p*-Hydroxyphenylacetate hydroxylase C1:reductase component	4HPA
A0O30_12110	*hpaI*	804	2,4-Dihydroxyhept-2-ene-1,7-dioic acid aldolase (EC 4.1.2.n4)	4HPA
A0O30_12115	*hpaH*	1308	4-Hydroxyphenylacetate symporter, major facilitator superfamily (MFS)	4HPA
A0O30_12120	*hpaF*	405	5-Carboxymethyl-2-hydroxymuconate delta-isomerase (EC 5.3.3.10)	4HPA
A0O30_12125	*hpaD*	924	3,4-Dihydroxyphenylacetate 2,3-dioxygenase (EC 1.13.11.15)	4HPA
A0O30_12130	*hpaE*	1470	5-Carboxymethyl-2-hydroxymuconate semialdehyde dehydrogenase (EC 1.2.1.60)	4HPA
A0O30_12135	*hpaG2*	765	5-Carboxymethyl-2-oxo-hex-3- ene-1,7-dioate decarboxylase (EC 4.1.1.68)/2-hydroxyhepta-2,4-diene-1,7-dioate isomerase (EC 5.3.3.-)	4HPA
A0O30_12140	*hpaG1*	660	5-Carboxymethyl-2-oxo-hex-3- ene-1,7-dioate decarboxylase (EC 4.1.1.68)/2-hydroxyhepta-2,4-diene-1,7-dioate isomerase (EC 5.3.3.-)	4HPA
A0O30_12145	*hpaR2*	906	Transcriptional activator of 4-hydroxyphenylacetate 3-monooxygenase operon, XylS/AraC family	4HPA

Table S1 *Cont.*

ORF [a]	Gene	Length (bp) [b]	Function [c]	Affiliated catabolic pathway [d]
A0O30_14370	*pcaG*	606	Protocatechuate 3,4-dioxygenase α chain (EC 1.13.11.3)	4HBA,VA
A0O30_14375	*pcaH*	720	Protocatechuate 3,4-dioxygenase β chain (EC 1.13.11.3)	4HBA,VA
A0O30_18255	*pobR*	879	Transcriptional regulator PobR, AraC family	4HBA
A0O30_18260	*pobA*	1188	4-Hydroxybenzoate 3-monooxygenase	4HBA
A0O30_19880	*antR*	972	Transcriptional regulator, AraC family	ANT
A0O30_19885	*antA*	1404	Anthranilate dioxygenase large subunit	ANT
A0O30_19890	*antB*	492	Anthranilate dioxygenase small subunit	ANT
A0O30_19895	*antC*	1032	Anthranilate dioxygenase reductase	ANT
A0O30_22000	*benR*	957	*BenABC* operon transcriptional activator BenR	BA, BFA
A0O30_22005		345	Hypothetical protein	
A0O30_22010	*benA*	1359	Benzoate 1,2-dioxygenase α subunit (EC 1.14.12.10)	BA, BFA
A0O30_22015	*benB*	486	Benzoate 1,2-dioxygenase β subunit (EC 1.14.12.10)	BA, BFA
A0O30_22020	*benC*	1011	Benzoate 1,2-dioxygenase, ferredoxin reductase component	BA, BFA
A0O30_22025	*benD*	762	1,2-Dihydroxycyclohexa-3,5-diene-1-carboxylate dehydrogenase (EC 1.3.1.25)	BA, BFA
A0O30_22030	*benK*	1329	Benzoate MFS transporter BenK	BA, BFA
A0O30_22035	*benL*	1200	Benzoate transport protein	BA, BFA
A0O30_22040	*benM*	1251	Benzoate-specific porin	BA, BFA
A0O30_22220	*paaN*	2055	Phenylacetic acid degradation protein PaaN, ring-opening aldehyde dehydrogenase (EC 1.2.1.3)	BA, BFA PAA
A0O30_22225	*paaM*	1233	Phenylacetic acid-specific porin PaaM	PAA
A0O30_22230		1563	Acetate permease ActP (cation/acetate symporter)	PAA
A0O30_22235	*paaL*	309	Putative membrane protein, clustering with ActP PaaL	PAA
A0O30_22240	*paaK*	1077	Phenylacetate-CoA oxygenase/reductase, PaaK subunit	PAA
A0O30_22245	*paaJ*	534	Phenylacetate-CoA oxygenase, PaaJ subunit	PAA
A0O30_22250	*paaI*	759	Phenylacetate-CoA oxygenase, PaaI subunit	PAA
A0O30_22255	*paaH*	282	Phenylacetate-CoA oxygenase, PaaH subunit	PAA
A0O30_22260	*paaG*	990	Phenylacetate-CoA oxygenase, PaaG subunit	PAA
A0O30_22265	*paaF*	1320	Phenylacetate-coenzyme A ligase (EC 6.2.1.30) PaaF	PAA
A0O30_22270	*paaE*	1221	Phenylacetic acid degradation protein PaaE, ketothiolase	PAA
A0O30_22275	*paaD*	441	Phenylacetic acid degradation protein PaaD, thioesterase	PAA
A0O30_22280	*paaC*	1518	3-Hydroxyacyl-CoA dehydrogenase PaaC (EC 1.1.1.-)	PAA

Table S1 *Cont.*

ORF [a]	Gene	Length (bp) [b]	Function [c]	Affiliated catabolic pathway [d]
A0O30_22285	*paaB*	792	Phenylacetate degradation enoyl-CoA hydratase PaaB (EC 4.2.1.17)	PAA
A0O30_22290	*paaA*	774	Phenylacetate degradation enoyl-CoA hydratase PaaA (EC 4.2.1.17)	PAA
A0O30_22295	*paaY*	600	Phenylacetic acid degradation protein PaaY	PAA
A0O30_22300	*paaX*	1005	Phenylacetic acid degradation operon negative regulatory protein PaaX	PAA
A0O30_24490	*vanK*	1332	Vannilate transporter VanK	VA
A0O30_24495	*bzfR*	897	LysR family transcriptional regulator	BFA
A0O30_24500	*bzfA*	1587	Benzoylformate carboxylase	BFA
A0O30_24505	*bzfB*	1341	BenK-like MFS transporter	BFA
A0O30_24510	*bzfC*	1476	Benzaldehyde dehydeogenase	BFA, VL
A0O30_24515	*bzfD*	1254	PhaK-like outer membrane porin	BFA
A0O30_24520	*vanR*	714	Transcriptional regulator	VA, VL
A0O30_24525	*vanA*	951	Vanillate *O*-demethylase oxidoreductase	VA, VL
A0O30_24535	*vanB*	1068	Vanillate *O*-demethylase oxygenase subunit	VA, VL
A0O30_24700	*catR*	873	Aromatic hydrocarbon utilization transcriptional regulator CatR (LysR family)	ANT, BA
A0O30_24705	*catC*	1122	Muconate cycloisomerase (EC 5.5.1.1)	ANT, BA
A0O30_24710	*catB*	291	Muconolactone isomerase (EC 5.3.3.4)	ANT, BA
A0O30_24715	*catA*	936	Catechol 1,2-dioxygenase (EC 1.13.11.1)	ANT, BA
A0O30_26225		1194	Putative *n*-hydroxybenzoate hydroxylase	4HBA
A0O30_26230	*hmgC*	642	Maleylacetoacetate isomerase (EC 5.2.1.2) @ Glutathione *S*-transferase, zeta (EC 2.5.1.18)	PHE, TYR
A0O30_26235		1353	4-Hydroxybenzoate transporter	4HBA
A0O30_26240	*hmgB*	699	Fumarylacetoacetase (EC 3.7.1.2)	PHE, TYR
A0O30_26245	*hmgA*	1056	Gentisate 1,2-dioxygenase (EC 1.13.11.4)	PHE, TYR
A0O30_26250	*hmgR*	944	Putative transcriptional regulator	PHE, TYR

[a,b] Refers to GenBank/ENA/DDBJ Accession No. NZ_LUVY00000000; [c] annotated by RAST or NCBI-PAGP.

[d] ANT.anthranilic acid catabolic pathway; BA, benzoic acid catabolic pathway; BFA, benzoylformic acid catabolic pathway; 4HBA, 4-hydroxybenzoic acid catabolic pathway; 4HPA, 4-hydroxypheylacetic acid catabolic pathway; PHE, phenylalanine catabolic pathway; TYR, tyrpsine catabolic pathway; VA, vanillic acid catabolic pathway; VL, vanillin catabolic pathway.

Table S2 List of data from GC/MS analysis

Peak	Retention time(min)	*m/z*	Products (trimethylsilylated)
1	6.2	77 105 135 179 194	Benzoic acid
2	8.4	73 105 135 178 207	Benzoylformic acid
3	5.3	65 135 165	Benzyl alchol
4	6.2	77 105 135 179 194	Benzoic acid
5	9.7	59 73 179 209 253 268 298	Vanillyl alcohol
6	10.7	73 126 223 253 267 297 312	Vanillic acid
7	9.6	73 179 209 253 268 298	Isovanillyl alcohol
8	10.1	73 126 223 253 267 297 312	Isovanillic acid
9	10.8	59 78 209 239 298 313 328	Syringic alcohol
10	11.7	78 141 253 297 312 327 342	Syringic acid
